# Prostate-Specific Membrane Antigen (PSMA) Theranostics for Treatment of Oligometastatic Prostate Cancer

**DOI:** 10.3390/ijms222212095

**Published:** 2021-11-09

**Authors:** Kristin A. Plichta, Stephen A. Graves, John M. Buatti

**Affiliations:** 1 Department of Radiation Oncology, University of Iowa, LL-W PFP, 200 Hawkins Dr., Iowa City, IA 52242, USA; stephen-a-graves@uiowa.edu (S.A.G.); john-buatti@uiowa.edu (J.M.B.); 2 Department of Radiology, University of Iowa, 3883 JPP, 200 Hawkins Dr., Iowa City, IA 52242, USA

**Keywords:** theranostics, PSMA, oligometastatic prostate cancer

## Abstract

Theranostics, a combination of therapy and diagnostics, is a field of personalized medicine involving the use of the same or similar radiopharmaceutical agents for the diagnosis and treatment of patients. Prostate-specific membrane antigen (PSMA) is a promising theranostic target for the treatment of prostate cancers. Diagnostic PSMA radiopharmaceuticals are currently used for staging and diagnosis of prostate cancers, and imaging can predict response to therapeutic PSMA radiopharmaceuticals. While mainly used in the setting of metastatic, castrate-resistant disease, clinical trials are investigating the use of PSMA-based therapy at earlier stages, including in hormone-sensitive or hormone-naïve prostate cancers, and in oligometastatic prostate cancers. This review explores the use of PSMA as a theranostic target and investigates the potential use of PSMA in earlier stage disease, including hormone-sensitive metastatic prostate cancer, and oligometastatic prostate cancer.

## 1. Introduction

Theranostics, a combination of therapy and diagnostics, is a field of medicine involving the use of radiopharmaceuticals to both diagnose and treat patients. A theranostic treatment both identifies suitable patients for treatment, provides a patient and cancer-specific treatment, and monitors response to treatment, all using the same or similar radiopharmaceuticals. Treatment typically involves the use of target-specific ligands that are bound to radioactive atoms. Depending on the physical properties of the radioisotope (half-life, emissions) and quantity of radiopharmaceutical administered to the patient (megabecquerel vs. gigabecquerel), these agents allow for diagnosis or subsequent disease treatment. While the term theranostics was only recently coined [1,2], the use of radiopharmaceuticals for this purpose has occurred since the 1940s with the imaging and treatment of hyperthyroidism and differentiated thyroid cancers with radioactive iodine [3]. In fact, the successful therapeutic application of radioactive iodine was the primary motivating factor leading to the development of the first nuclear imaging system, the rectilinear scanner [4]. Radioactive isotopes of iodine allow for both imaging (^123^I, ^124^I, ^131^I) of iodine uptake in the thyroid and potential metastatic sites, and treatment of the thyroid gland itself or disease from differentiated thyroid cancers with oral or intravenous ^131^I-NaI administration [3]. Other theranostic agents currently in use include ^177^Lu-DOTATATE (paired with Gallium-68-DOTATATE or DOTATOC PET/CT imaging) for the treatment of metastatic neuroendocrine tumors [5]; Radium-223-dichloride (paired with ^99m^Tc-methylene diphosphonate bone scans or ^18^F-NaF PET/CT scans) for the treatment of metastatic, castrate-resistant prostate cancers with bone metastases [6], and ^131^I-iobenguane (^131^I-MIBG; paired with ^123^I-MIBG) for the treatment of metastatic paragangliogliomas, pheochromocytomas, and neuroblastomas [7,8,9]. As a discipline, theranostics aims to identify appropriate candidates for treatment, provide therapy, and monitor response, thereby allowing for a more personalized approach.

While some radiometals directly target disease sites such as uptake of radioactive iodine within thyroid tissue, radiopharmaceuticals can also be designed to target cancer-specific receptors. The ideal theranostic target is both overexpressed in cancer cells and has minimal or no expression in normal tissues, thereby permitting high tumor dose with low toxicity to normal tissues. These agents typically have three distinct parts: a radionuclide (often conjugated by a chelator), a linker, and a ligand that binds with a targeted cancer cell receptor [10,11]. The use of a linker between the radionuclide and ligand prevents loss of molecular recognition (also referred to as binding affinity) by a reduction in steric hindrance. Furthermore, linkers can be used to modify the pharmacokinetic properties of the overall radiopharmaceutical by increasing or decreasing net charge and lipophilicity [12]. While some radiopharmaceuticals can be used for both diagnostic imaging and therapeutic treatment (^131^I), treatment often involves a theranostic product pair. A theranostic product pair consists of two agents with the same targeting ligand, but different radionuclides. One part of the pair is specific for diagnostic imaging (predominantly gamma-ray emission suitable for SPECT or PET), while the other is specific for treatment (predominantly by alpha or beta particle emission). Thus, the theranostic product pair works together to identify suitable patients for treatment (diagnostic agent) or treat the patient (therapeutic agent) [10,11].

## 2. Prostate-Specific Membrane Antigen as a Theranostic Target

Prostate-specific membrane antigen (PSMA) is a promising theranostic treatment target. PSMA is a 750 amino acid type 2 integral membrane glycoprotein with two monomers and an intracellular, transmembrane, and extracellular domain [13]. The discovery of PSMA was enabled by many researchers working in the 1970s and 1980s to develop a stable in vitro cell line of human prostate carcinoma, while preserving several key characteristics, including functional differentiation, malignant properties, possessing an aneuploid male karyotype, and expression of a high-affinity androgen receptor. The LNCaP cell line—established in 1983 by Horoszewicz et al. [14]—was used for the development of a monoclonal mouse antibody (7E11-C5) to the prostate cancer cell membrane [15]. This antibody was subsequently used to identify the DNA that encoded PSMA [16], which allowed for the evaluation of PSMA expression levels in various human tissues. While PSMA is expressed at low levels in normal prostate epithelium, it is overexpressed (up to 1000 times higher) in 90–95% of prostate cancers [17]. In contrast to benign prostatic epithelium where PSMA resides in the cytoplasm, PSMA is in the luminal epithelium of prostatic ducts in prostate cancers and presents a large extracellular binding domain [17,18]. PSMA expression is positively correlated with more aggressive disease, including high PSA, high Gleason scores, and early recurrence [17,18,19]. The highest levels of PSMA expression are found in metastatic and castrate-resistant disease [17,19]. The expression of PSMA is not limited to prostate cancers, as PSMA is found in the tumor neovasculature of some bladder, pancreas, lung, and thyroid cancers [17]. Although PSMA is also expressed in other normal tissues including the lacrimal glands, salivary glands, proximal renal tubules, epididymis, ovary, proximal small bowel, and neurogenic crypt cells, expression is at much lower levels than what is seen in prostate cancer [17,18]. PSMA is internalized after extracellular receptor binding via endocytosis of clathrin-coated pits [20]. The differential expression of PSMA between normal tissues and cancer cells, and the ability for PSMA to be internalized after binding antibodies or targeted small molecules, make PSMA an attractive target for a theranostic treatment.

**Figure 1:**
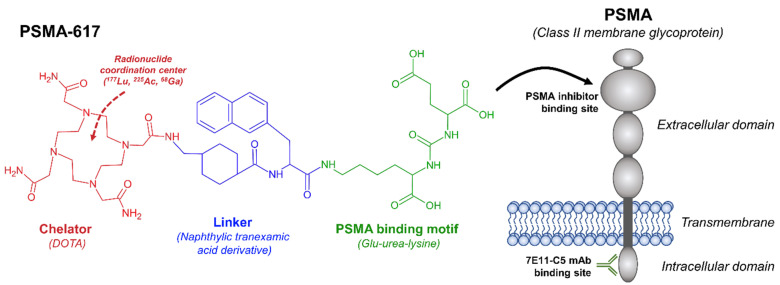
Structure of PSMA-617 with main components (chelator, linker, bonding motif) highlighted. PSMA-617, as well as other small-molecule PSMA inhibitors (DCFPyL, PSMA-11, PSMA-I&T), are targeted to the extracellular domain of PSMA, whereas ^111^In-7E11-C5 (ProstaScint^®^) is now known to bind to the intracellular domain.

Initial diagnostic imaging with PSMA used the same 7E11-C5 antibody (capromab pendetide), labeled with indium-111, and branded under the name ProstaScint^®^ [21]. Although initially promising, capromab pendetide targets an intracellular domain of PSMA, limiting its utility as a diagnostic tool [22]. More recent PSMA imaging agents have been developed with lower molecular weight, thereby increasing blood clearance rate, and targeted to the extracellular binding domain of PSMA (illustrated in Figure 1). These changes have resulted in greatly improved image quality and increased accuracy in diagnosing disease. Positron-emitting radioisotopes (i.e., ^18^F and ^68^Ga) have also been favored recently, enabling high-resolution and quantitative PET/CT imaging. Currently, the most common diagnostic agents are ^68^Ga-PSMA-11 and ^18^F-DCFPyl (approved by FDA in 2020 and 2021, respectively). These agents can localize areas of disease recurrence at very low levels of PSA, with tumor avidity being observed in the majority of patients with prostate cancer [23,24,25]. PSMA PET/CT scans have increased diagnostic accuracy in initial staging and in investigating disease recurrence in comparison to standard imaging with CT and MRI. A recent phase 3 clinical trial (proPSMA trial) showed that ^Ga^68 PSMA-11 PET/CT scans were more accurate in detecting lymph nodal and distant metastases than conventional imaging with a bone scan and a contrast-enhanced CT of the abdomen and pelvis for patients with newly diagnosed high-risk prostate cancer [23]. Likewise, the CONDOR and OSPREY clinical trials have demonstrated high sensitivity for detecting distant metastatic prostate cancer, high specificity for detecting pelvic lymph node metastases in the setting of high-risk disease, and a high rate of correct localization rate of lesions (percentage of patients with a one to one correspondence between at least one lesion identified on ^18^F-DCPyl-PET/CT scans and the lesion identified by central readers or pathology) in the setting of biochemical recurrence [24,25]. Preliminary studies have also shown that PSMA imaging can significantly change a patient’s treatment plan in the high risk and biochemical recurrence setting, and there is now an ongoing phase 3 trial investigating whether the impact of PSMA imaging on salvage radiation therapy treatment planning will result in improved clinical outcomes [26].

Several radionuclides have been utilized for the therapeutic use of PSMA, including Lutetium-177, Iodine-131, and Actinium-225. The most common therapeutic nuclide in use (^177^Lu) decays by beta emission (100%) with a half-life of 6.6 days [27,28], followed by gamma emission (113 keV, 6.2%; 208 keV, 10.4%) resulting from relaxation of the residual daughter ^177^Hf nucleus. The beta decay of ^177^Lu is relatively low energy (496 keV endpoint, 137 keV mean), resulting in a mean range of 0.7 mm and a maximum range of 2.1 mm in soft tissues [29]. Given the relatively long half-life and short tissue penetration, ^177^Lu allows for efficient delivery of therapeutic radiation to prostate cancer lesions. Although not particularly useful for therapy, the gamma emissions resulting from the decay of ^177^Lu allow for high-quality SPECT/CT imaging, thereby enabling image-based dosimetry following administration of the therapeutic radiopharmaceutical. These therapies tend to be given in multiple doses (4–6 treatments at 6–8 weeks intervals), which means that post-treatment dosimetry allows for adaptation in subsequent cycles of therapy based on absorbed dose to critical structures and tumor tissues [30,31]. In contrast to ^177^Lu and ^131^I, ^225^Ac decays by alpha emission with a half-life of 10 days. Preliminary studies have shown potentially greater antitumor efficacy with reduced bone marrow toxicity with PSMA-targeted ^225^Ac agents, likely due to the short range associated with alpha particles (0.04–0.06 mm) [32]. In terms of therapeutic ligand/vectors, small molecular inhibitors of PSMA are predominantly used as radioligands, due to concerns about increased hematologic toxicity from prolonged blood retention of monoclonal antibodies [33]. Multiple PSMA therapeutic ligands have been investigated, however, the currently leading candidate is ^177^Lu-PSMA-617 which carries the chelator DOTA for conjugation of various radiometals [34].

Most clinical trials have focused on the use of PSMA radiotherapeutics for metastatic, castrate-resistant prostate cancers (MCRPC). The reason for the selection of this patient population is twofold: first, there is a current need for durable treatment options in this patient population, as chemotherapy and other systemic therapy responses are typically limited in duration, and second, PSMA expression is typically very high in this patient population. Initial trials with ^177^Lu-based PSMA radioligands have shown promising results, including significant PSA declines and improvement in symptoms with PSMA. A meta-analysis of ^177^Lu-PSMA studies in MCRPC showed that 46% of patients had a greater than 50% PSA decline after treatment with Lu-PSMA, and 75% of patients had any PSA decline after therapy [34]. In the phase 2 TheraP trial, treatment with ^177^Lu-PSMA-617 in comparison with cabazitaxel resulted in higher PSA responses and fewer grade 3 or 4 adverse responses [35]. The recently published phase 3 VISION trial randomizing patients (n = 831) with MCRPC with PSMA-positive cancers with disease progression after prior treatment with at least one androgen-receptor pathway inhibitor and taxane chemotherapy regimen to ^177^Lu-PSMA-617 or standard of care treatment demonstrated statistically significant improvements in progression-free survival (8.7 vs. 3.4 months) and overall survival (15.3 vs. 11.3 months) with ^177^Lu-PSMA-617 treatment [36]. The positive results from the VISION trial confirmed the benefit of ^177^Lu-PSMA-617 in the MCRPC setting, and this agent is currently under review by the FDA. In terms of treatment with alpha emitters, preliminary studies of ^225^Ac-PSMA-617 have also shown high response rates in terms of PSA declines both as an initial treatment and with progression following treatment with ^177^Lu-PSMA-617 [32,37,38,39]. It is important to note that both the TheraP trial and the VISION trial screened patients based on PSMA expression via ^68^Ga-PSMA-11 scans (and in the case of the TheraP trial, no metastatic sites with discordant ^18^F-FDG-positive and PSMA-negative findings), thus allowing for selection of patients who are likely to respond to potential ^177^Lu-PSMA-617 therapy [35,36].

PSMA-targeted radioligands have a favorable toxicity profile. Common side effects include dry mouth or xerostomia (up to 30% of men), fatigue, and nausea, which correlate with areas of PSMA expression in normal tissues [34,35,36,40]. Hematologic toxicity has also been reported, and typically occurs in patients with extensive bone metastases and borderline marrow function, likely resulting from a combination of factors, including reduced functional marrow reserve, increased dose per administered activity due to elevated photon-based irradiation of the bone marrow, as well as treating metastases near the marrow itself [35,36,41]. ^225^Actinium agents have demonstrated decreased hematologic toxicity, but higher rates of xerostomia [32]. PSMA ligands do bind to the kidneys, and the radiopharmaceuticals are predominantly renally excreted due to their low molecular weight and hydrophilic nature [42]. There have been no reports of grade 3 or 4 renal toxicity with the use of PSMA radioligands in acute or subacute settings [43,44]. If PSMA radioligands are utilized in the treatment of prostate cancer at earlier stages, further follow-up will necessary to determine potential long-term (over 2 years) toxicity after therapy, as other small peptide radionuclides have long-term kidney effects [45].

While the focus of the majority of PSMA radioligand studies is in patients with MCRPC, high PSMA expression is also found in earlier disease stages, including in the high risk, recurrent, and oligometastatic settings. The traditional treatment of metastatic castrate-sensitive prostate cancer is the initiation of androgen deprivation, potentially in combination with chemotherapy or an androgen receptor inhibitor [46,47]. However, a significant number of patients will progress to MCRPC, where there are fewer treatment options. The use of PSMA-targeted radiopharmaceuticals at earlier stages may prevent progression in these patients or potentially allow for longer periods of time without androgen deprivation therapy. There are two ongoing clinical trials examining the use of ^177^-Lu-PSMA-617 in the metastatic, hormone-sensitive (or hormone naïve) population [48,49]. The UpFrontPSMA trial is investigating the use of docetaxel in combination with ^177^Lu-PSMA-617 as compared to docetaxel alone in men with a new diagnosis of high-volume metastatic hormone-naïve prostate cancer [48]. A large-scale, phase 3 trial (sponsored by Novartis) is investigating the use of ^177^Lu-PSMA-617 in combination with standard of care treatment (androgen deprivation therapy and androgen receptor-directed therapy) compared to standard of care treatment alone is also recruiting participants [49]. These trials will provide insight into potentially combining treatments for the metastatic, hormone-sensitive patient population [48,49].

## 3. PSMA Radiopharmaceutical treatment in Oligometastatic Disease

In the setting of oligometastatic disease, it has been proposed that definitively treating patients with radiation therapy or surgery to the prostate or metastatic sites could improve overall survival and decrease or delay androgen deprivation therapy [50,51]. In addition, as treatment with androgen deprivation can result in unwanted adverse side effects—including sexual dysfunction, weight gain, and cardiac risks—there is a need to identify potential treatment options to delay or reduce the need for androgen deprivation therapy. In the STAMPEDE trial, radiation therapy to the prostate improved overall survival in patients with low metastatic burden (no visceral metastases, four or fewer bone metastases with no more than one lesion beyond the vertebral body or pelvis), based on a prespecified subset analysis [52]. In the setting of recurrent prostate cancer with oligometastatic disease, definitive treatment of metastatic sites can improve progression-free survival and delay the initiation of androgen deprivation [50,51]. In the phase 2 STOMP trial, 62 patients with recurrent prostate cancer with one to three metastases identified on an ^11^C-choline PET/CT scan were randomized to surveillance or metastatic lesion-directed therapy (surgery or stereotactic body radiotherapy (SBRT) to all metastatic lesions) [50]. The median androgen deprivation-free survival of the metastatic-directed therapy group was 21 months, in comparison to 13 months in the surveillance group, with low rates of toxicity [50]. Likewise, the phase II ORIOLE trial compared SBRT to observation for men with recurrent, hormone-sensitive prostate cancer with one to three metastatic lesions identified on PSMA PET/CT scans, and found improvement in median progression-free survival (not reached vs 5.8 months) and biochemical progression-free survival with SBRT in comparison to observation [51].

While definitive treatment of metastatic lesions by SBRT or metastasectomy in the oligometastatic setting can improve cancer-related outcomes, not all sites of metastases are in locations amenable to SBRT or surgical resection. In addition, while very small lesions may be seen on PSMA PET/CT scans, these lesions may not be feasibly targeted with SBRT due to their small size or difficulty ascertaining their location without the radiotracer. Treatment with PMSA-targeted radioligands could therefore be beneficial in this setting, by treating all lesions (both those seen on PET imaging, and those too small to be seen). Given the decay characteristics of ^177^Lu, including the relatively short-range beta emissions, it is likely that ^177^Lu can effectively target small metastatic lesions [29].

While there are few clinical trials investigating the use of theranostic PSMA agents in the setting of oligometastatic disease, these studies do showcase the potential promise of this agent in these settings (Table 1). Von Eyben et al. reported the results of a patient treated with ^177^Lu-PSMA-617 after the third recurrence of castrate-resistant prostate cancer in a pelvic lymph node [53]. The patient had previously received 78 Gy to an initial pelvic lymph node, with a partial response. The patient then received 60 Gy to a retroperitoneal lymph node, with a response. Repeat ^68^Ga-PSMA-11 imaging revealed new uptake in a mediastinal lymph node and several infradiaphragmatic lymph nodes. He was treated with two administrations of 6 GBq of ^177^Lu-PSMA-617 (substantially less than the cumulative 29.6–44.4 GBq administered in the VISION trial), resulting in no detectable PSA for 5 months, and a slight rise in PSA following. A small pilot study of 10 patients with hormone-sensitive prostate cancer with fewer than 10 metastatic lesions (with no visceral metastatic disease), no curable treatment options (surgery or external beam radiation therapy), and tumor PSMA uptake higher than liver investigating the use of ^177^Lu-PSMA-617 showed promising results with response in terms of PSA decline [54]. All patients had stabilization of PSA velocity after two cycles, and 5/10 patients showed a PSA decline of >50%. After 24 weeks (the study observation period), PSA was still decreasing in three patients, with one patient having a biochemical complete response. There were no grade 3–4 toxicities, and the observed grade 1–2 toxicities subsided within a few weeks [55]. The absorbed dose from therapy with ^177^Lu-PSMA-617 was found to be comparable to that seen in the treatment of high-volume MCRPC based on dosimetric analysis of tumor and normal tissues (kidney, bone marrow, liver, and salivary gland) [55]. In addition, a high tumor to organ ratio of ^177^Lu-PSMA-617 was observed [55]. There is a current phase 2 trial investigating the use of ^177^Lu-PSMA-617 in the setting of oligometastatic disease currently recruiting patients [56,57].

Of note, in addition to the oligometastatic setting, PSMA-targeted radiopharmaceuticals are also being investigated in the setting of high risk or intermediate risk localized prostate cancer with high ^68^Ga-PSMA expression [58].

Early studies investigating the relationship between tumor radiation absorbed dose from ^177^Lu-PSMA therapy and treatment response have demonstrated an increased likelihood of response with increasing dose to tumors [59,60]. The capacity to perform personalized image-based dosimetry [61] in patients treated with PSMA-targeted agents may prove critical in the management of patients with oligometastatic disease. Organs at risk from PSMA-targeted radioligand therapies do not typically overlap with those encountered in external-beam radiotherapy-based treatment of metastatic disease. Therefore, strategies combining radiation modalities may lead to improved patient outcomes in situations where radiopharmaceutical dosimetry indicates that additional radiation doses may be needed to achieve a favorable treatment response. Further work is needed to characterize these dose–response relationships, as well as human trials demonstrating the safety of combination-based therapy.

## 4. Conclusions

With the combination of diagnostic imaging and therapeutic treatment, theranostics allows for the selection of patients most likely to benefit from potential treatment. PSMA is a promising theranostic biomarker for the detection and treatment of prostate cancers. While the benefit of treatment has been mainly investigated in the setting of metastatic, castrate-resistant prostate cancers, PSMA-targeted radiopharmaceuticals may also be beneficial in a hormone-sensitive, metastatic prostate cancer setting, and with oligometastatic disease. Ongoing research studies will help to clarify the benefit of radiopharmaceuticals in an oligometastatic disease setting.

## Figures and Tables

**Table 1:** ^177^Lu-PSMA-617 in metastatic, hormone-sensitive prostate cancers.

Ref.	Patient Population	Study Type	Cycles of Therapy	Patient Numbers	Status	Results
Von Eyben et al. [53]	MCRPC, ^68^Ga-PSMA-PET-positive disease, limited to lymph nodes	Case Report	2	1	Completed	No detectable PSA for 5 months, Slight rise in PSA at 5 months
Prive et al. [54]	Hormone-sensitive, ^68^Ga-PSMA-PET positive disease, maximum 10 metastases (no visceral metastasis)	Pilot Study	2	10	Completed	Stabilization of PSA velocity (10/10), PSA decline > 50% (5/10), PSA decline after 24 weeks (3/10), Biochemical complete response (1/10)
Prive et al. [56,57]	Biochemical recurrence, no prior hormonal therapy, ^18^F-PSMA PET/CT-positive disease in bones and/or lymph nodes, maximum 5 metastases	Phase 2	2	58 (planned)	Recruiting	Pending
Azad et al. [48]	Hormone-naïve ^68^Ga-PSMA-PET-positive high volume metastatic disease	Phase 2	2	140 (planned)	Recruiting	Pending
Novartis [49]	Hormone-sensitive, Hormone-naïve (or minimally treated), ^68^Ga-PSMA-PET positive disease with bone, visceral or lymph node metastases	Phase 3, randomized, crossover	6	1126 (planned)	Recruiting	Pending

## Data Availability

Not applicable.
